# Impact of Sodium Butyrate Treatment in LPS-Stimulated Peripheral Blood Mononuclear Cells of Poorly Controlled Type 2 DM

**DOI:** 10.3389/fendo.2021.652942

**Published:** 2021-07-29

**Authors:** Heri Wibowo, Dante S. Harbuwono, Dicky L. Tahapary, Rona Kartika, Saraswati Pradipta, Rahma A. Larasati

**Affiliations:** ^1^Department of Parasitology, Faculty of Medicine, Universitas Indonesia, Jakarta, Indonesia; ^2^Division of Metabolic Endocrinology and Diabetes, Department of Internal Medicine, Faculty of Medicine, Universitas Indonesia, Jakarta, Indonesia; ^3^Integrated Laboratory, Faculty of Medicine, Universitas Indonesia, Jakarta, Indonesia; ^4^Department of Biomedicines, Faculty of Medicine, Universitas Muhammadiyah Jakarta, Jakarta, Indonesia

**Keywords:** poorly controlled type 2 diabetes mellitus, inflammatory response, peripheral blood mononuclear cells, butyrate, lipopolysaccharide (LPS)

## Abstract

Type 2 diabetes mellitus (T2DM) is associated with chronic low-grade inflammation, which is marked by the dysregulation of innate and adaptive immune responses. Therefore, reducing inflammation, possibly through an immunoregulatory agent, may play a role in T2DM treatment. Butyrate is the most potent short-chain fatty acid (SCFA), and it exerts anti-inflammatory properties by inhibiting histone deacetylase activity. As an immunoregulatory agent, sodium butyrate can inhibit nuclear factor kB (NF-kB) activation and reduce the production of pro-inflammatory cytokines in immune cells. The aim of the study was to measure the level of plasma butyrate in poorly controlled T2DM and normoglycemic participants and to compare the response of peripheral blood mononuclear cells (PBMCs) to sodium butyrate treatment between the groups by measuring production of the following cytokines: tumor necrosis factor (TNF)-α, interleukin (IL)-6, interferon (IFN)-γ, IL-13, and IL-10. The *in vitro* study examined the PBMCs of 15 participants with poorly controlled T2DM and 15 normoglycemic participants. PBMCs were cultured with the following stimulations for two days at a temperature of 37°C and 5% CO_2_: 100 ng/mL lipopolysaccharide (LPS), 1 mM sodium butyrate, or a combination of 100 ng/mL LPS and 1 mM sodium butyrate. Plasma butyrate was measured using gas chromatography-mass spectrometry, and cytokines from culture supernatant were analyzed using magnetic beads multiplex assay. Plasma butyrate levels in participants with poorly controlled T2DM did not significantly differ from those in normoglycemic participants (*p* = 0.105). Compared to treatment with an LPS-stimulated PBMC culture, treatment with 1 mM sodium butyrate reduced the levels of TNF-α (*p < 0.039*) and IFN-γ (p < 0.038) in normoglycemic participants. The same general trend was seen in PBMC from participants with poorly controlled T2DM, but higher variability appeared to preclude statistical significance. These data suggest that butyrate may modulate inflammatory cytokine production in human PBMCs, but more research is needed to determine if butyrate is anti-inflammatory in poorly controlled T2DM.

## Introduction

Gut microbiota have an essential function in maintaining intestinal homeostasis and human health by regulating the immune system, maintaining epithelial barriers, and protecting against several diseases (1). Among the numerous metabolites produced by commensal gut bacteria, short-chain fatty acids (SCFAs) have received the most attention in the context of alleviating host health conditions. SCFAs are the end products of fermentation contained carbolic acids with aliphatic tails. SCFAs are found in high concentrations in the gut lumen, and they can be absorbed *via* non-ionic diffusion across colonic epithelial cells (2).

In addition to being the major energy source for colonocytes (3), SCFAs have long been known to modulate the immune response. Among SCFAs, butyrate is the main type that exerts anti-inflammatory properties (4). Butyrate affects neutrophil function and migration (5) and inhibits nuclear factor kappa B (NF-κB), tumor necrosis factor (TNF)-α, and interleukin (IL)-1β (6). In addition, butyrate reduces the adherence of monocytes or lymphocytes to cytokine-stimulated endothelial cells (7) and inhibits interferon-γ signaling (8). Butyrate probably regulates the immune system by modifying cellular processes, such as promoting the activation of G-protein coupled receptors (GPCRs), inhibiting histone deacetylase (HDAC), and stimulating histone acetyltransferase (4). Correa-Oliveira et al. (4) demonstrated that butyrate could hinder the development of murine bone marrow-derived macrophages, suppress the activation of T cells, and induce apoptosis. In Crohn’s disease, butyrate has been known to reduce TNF production and inhibit NF-κB activation in lamina propria cells and peripheral blood mononuclear cells (PBMCs) (9).

Type 2 diabetes mellitus (T2DM) is a metabolic disease characterized by hyperglycemia and insulin resistance (10, 11) and is often associated with low-grade inflammatory conditions (12). In addition, T2DM is correlated with adaptive immune dysfunction, which is assessed by T helper (Th)-1/Th-2 imbalance (12). Our previous study showed that PBMCs from T2DM patients demonstrated an enhanced cellular responsiveness to phytohemagglutinin (PHA), marked by lower interferon (IFN)-γ production, lower indoleamine 2,3 dioxygenase production, and higher TNF-α/IFN-γ and IL-6/IFN-γ ratios compared to normoglycemic participants (13, 14).

Treatment with SCFAs, particularly butyrate, is thought to be beneficial for patients with low-grade inflammatory conditions such as T2DM, but data concerning the effect of butyrate on reducing cytokine production from PBMC in T2DM patients are limited. Sodium butyrate was found to ameliorate insulin resistance and decrease plasma glucose levels in diabetic rats (15). In addition, Larasari et al. (16) showed that the short-term butyrate treatment reduced the accumulated distance of monocyte migration and decreased the inflammatory potential released by monocytes in healthy subjects and in T2DM patients. In the current study, participants’ levels of plasma butyrate were measured, and the responses of peripheral blood mononuclear cells (PBMCs) to sodium butyrate were compared between poorly controlled T2DM patients and normoglycemic participants in the presence of lipopolysaccharide; this comparison was made by measuring cytokine production from innate immunity (TNF-α and IL-6) and Th1/Th2 cytokine balance (IFN-γ, IL-13, and IL-10).

## Materials and Methods

### Participants

The *in vitro* study was performed on PBMCs collected from 15 normoglycemic participants and 15 participants with poorly controlled T2DM. In our former study of T2DM, the sample size of participants with T2DM was 21 people. In this study, we excluded six of those participants, whose T2DM was controlled. Therefore, only 15 participants were included in this analysis.

The normoglycemic participants were 30–55-year-olds with fasting blood glucose (FBG) < 100 mg/dL and glycated hemoglobin (HbA1c) < 5.7% who had never been diagnosed with T2DM by a physician. The criteria for poorly controlled T2DM patients, according to a glycemic target from the American Diabetes Association (17), are people 30–55 years of age, with FBG > 130 mg/dL, and/or HbA1c > 7%. The exclusion criteria for both groups were current pregnancy, steroid use, use of non-steroidal anti-inflammatory drugs or antibiotics within the two weeks preceding the study, and infection at the time of blood collection, including symptoms of fever, sore throat, respiratory tract infection, or urinary tract infection.

The study was approved by the ethical committee of the Faculty of Medicine of Universitas Indonesia, reference number 18-03-0236. All participants provided their written informed consent prior to taking part in this research.

### Plasma Butyrate Analysis

Plasma butyrate was analyzed using Agilent 5973 mass spectrometry with 6890 Plus gas chromatography system (Agilent Technologies, Santa Clara, CA, USA). In brief, 1 mL of plasma was added to 3 mL of 5% formic acid and 2.5 mL of ethyl acetate. The suspension was homogenized for 30 minutes and centrifuged at 15000 rpm for 5 minutes. Six mL of supernatant was isolated, and 0.1 g sodium sulfate anhydrous was added. Then, the solution was injected into the gas chromatography–mass spectrometry machine.

### Peripheral Blood Mononuclear Cells (PBMC) Isolation and Culture

PBMCs were isolated by centrifugation from Roswell Park Memorial Institute (RPMI) 1640-diluted blood through Ficoll-Paque Plus (GE Healthcare Life Sciences, Chicago, IL, USA). Then, PBMCs were resuspended in a cell culture medium consisting of RPMI 1640 supplemented with 10% heat-inactivated fetal bovine serum and 1% penicillin-streptomycin at a concentration of 10^6^ cells/mL. A 500 µL cell suspension was incubated in a 24-well tissue culture plate for one day at a temperature of 37°C with 5% CO2. Then, either 500 µL of 200 ng/mL LPS (Sigma Aldrich, St. Louis, MO, USA), 500 µL of 2 mM sodium butyrate (Sigma Aldrich), 500 µL of 200 ng/mL LPS and 2 mM sodium butyrate, or 500 µL of cell culture medium (the unstimulated group) were added to the wells. Then, the culture plates were incubated for 48 h at a temperature of 37°C with 5% CO2. On day three, cell culture supernatants were harvested and stored at a temperature of –80°C.

### Cytokines Assay

Cell culture supernatants were measured for *in vitro* cytokine production. TNF-α, IL-6, IFN-γ, IL-13, and IL-10 were measured using bead-based multiplex assay (R&D Systems, Minneapolis, MN, USA) as described in a previous paper (13). The detection limits were 0.6 pg/mL for TNF-α; 1.11 pg/mL for interleukin-6 (IL-6); 1.27 pg/mL for interferon-γ (IFN- γ); 2.01 pg/mL for interleukin-13 (IL-13); and 0.3 pg/mL for interleukin-10 (IL-10).

### Data Analysis

Data were analyzed using IBM SPSS Statistics, Version 23.0 (IBM, Armonk, NY, USA). A Shapiro–Wilk test was used to assess normality between groups. Since the baseline characteristics were normally distributed, unpaired Student’s *t*-tests were used to compare the baseline characteristics of the two groups. Plasma butyrate concentration and cytokine production were not normally distributed. Therefore, the Mann–Whitney *U*-test was used to compare the plasma butyrate concentrations of the two groups, and the Kruskal–Wallis test followed by Bonferroni adjustment were used to conduct six comparisons of the differences in cytokine production among the four stimulation groups: LPS, LPS and butyrate, butyrate, and unstimulated culture. Because they were not normally distributed, data of plasma butyrate concentration and cytokines’ production were presented as medians (minimum-maximum). GraphPad Prism 8 was used to create graphs and data visualization. The data were considered statistically significant if the *p*-value was < 0.05.

## Results

### Subject Characteristics

The baseline characteristics of the 15 normoglycemic participants and 15 participants with poorly controlled T2DM enrolled in this study are presented in **Table 1**. As expected, the poorly controlled T2DM group had higher FBG and HbA1c with a mean ± standard deviation of 205.53 ± 99.59 mg/dL and 9.38 ± 2.95%, respectively. Since this study did not match the age and body mass index (BMI) of participants, the normoglycemic group was younger and leaner than the T2DM group, which introduced a high chance of confounding factors; thus, we included age and BMI as covariates for further analysis.

**Table 1:** Baseline subject characteristics.

Baseline subject characteristics	Normoglycemic group (*n* = 15)	Poorly controlled T2DM group (*n* = 15)	*p*-value
Female sex (*n*, %)	10 (66.7)	8 (55.6)	0.355
Male sex (*n*, %)	5 (33.3)	7 (44.4)	
Age (years)	39.73 ± 4.52	47.33 ± 6.55	0.001**
BMI (kg/m^2^)	22.81 ± 2.78	26.80 ± 2.81	0.001**
FBG (mg/dL)	89.51 ± 11.84	205.53 ± 99.59	<0.001***
HbA1c (%)	4.87 ± 0.52	9.38 ± 2.95	<0.001***

T2DM, type 2 diabetes mellitus; BMI, body mass index; FBG, fasting blood glucose; HbA1c, glycosylated hemoglobin; **p < 0.01, ***p < 0.001. Age, BMI, FBG, and HbA1c were presented as mean ± standard deviation, and statistical significances were calculated using unpaired Student’s t-tests. The percentage of participants of each sex was calculated using Fisher’s exact test.

### Plasma Butyrate in T2DM Patients

**Figure 1:**
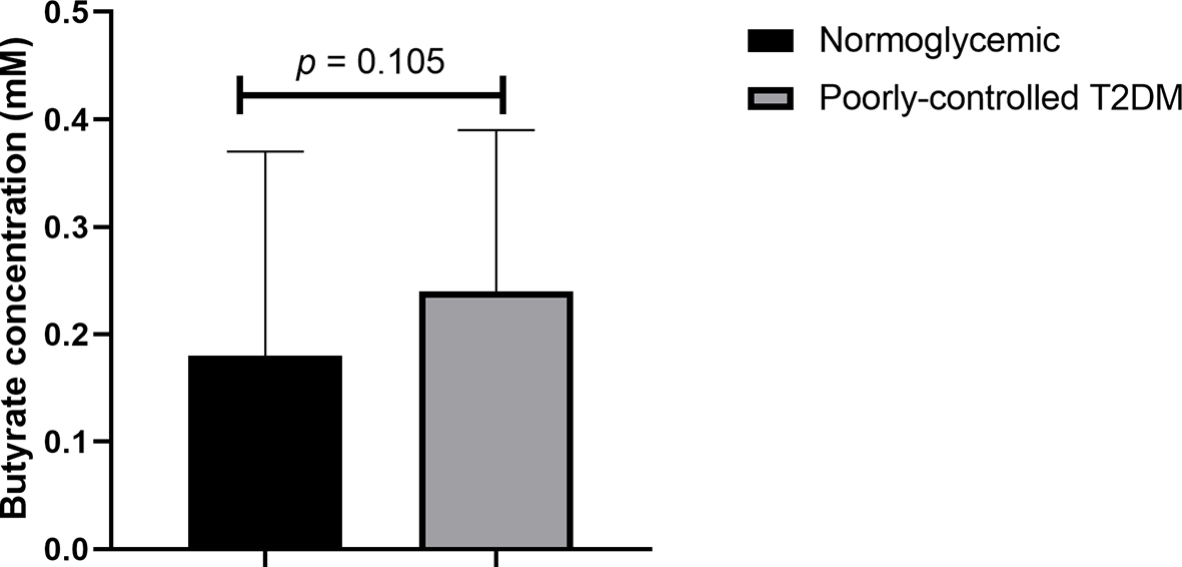
Comparison of plasma butyrate concentration of poorly controlled T2DM patients and normoglycemic participants. T2DM, type 2 diabetes mellitus. The comparison was assessed using the Mann–Whitney test.

As shown in **Figure 1**, even after age and BMI adjustment, plasma butyrate levels of participants with poorly controlled T2DM did not significantly differ from normoglycemic participants [median 0.24 (0.14 – 0.78) *vs* 0.18 (0.12 – 0.69) mM, *p* = 0.105].

### Cytokine Concentration in PBMC Culture Supernatants

**Figure 2:**
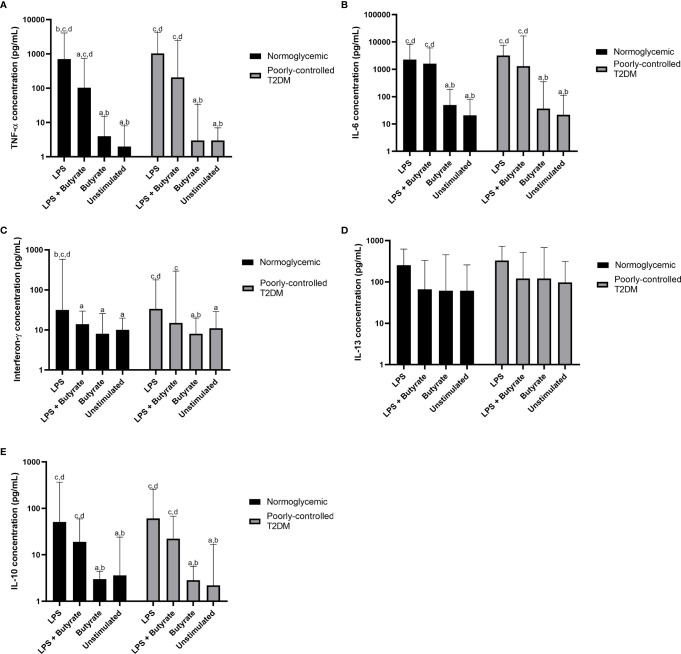
Comparisons of the production of **(A)** TNF-α, **(B)** IL-6, **(C)** interferon-γ, **(D)** IL-13, and **(E)** IL-10 among LPS, LPS + butyrate, butyrate stimulated PBMC cultures, and unstimulated PBMC cultures. T2DM, type 2 DM; TNF-α, tumor necrosis factor-α; IL-6, interleukin-6; IL-13, interleukin-13; IL-10, interleukin 10; LPS, lipopolysaccharide at total concentration of 100 ng/mL; LPS + butyrate, lipopolysaccharide at total concentration of 100 ng/mL and sodium butyrate at total concentration of 1 mM; and butyrate, sodium butyrate stimulation at total concentration of 1 mM. The comparisons were assessed using the Kruskal–Wallis test with a *post hoc* pairwise comparisons. The data were presented as median ± range. a: *p* < 0.05 *vs* LPS; b: *p* < 0.05 *vs* LPS + butyrate; c: *p* < 0.05 *vs* butyrate; d: *p* < 0.05 *vs* unstimulated.

According to the Kruskal–Wallis test (**Table 2**), the production of TNF-α, IL-6, IFN-γ, and IL-10 were different in participants with poorly controlled T2DM and normoglycemic participants. However, there was no difference in the cytokine production of PBMC cultures between poorly controlled T2DM and normoglycemic participants (**Supplemental Table 1**). *Post hoc* testing found that TNF-α, IL-6, IFN-γ, and IL-10 were more greatly increased with LPS stimulation alone compared to butyrate stimulation and no stimulation in both normoglycemic and poorly controlled T2DM participants (**Figure 2**).

**Table 2:** Kruskal–Wallis test to compare the cytokine production of various stimulated PBMC cultures in the study groups.

Cytokines	Stimulants	n	Median (minimum-maximum)	H statistics	df	*p-*value
Normoglycemic subject						
TNF-α	LPS	15	707 (73 – 4071)	45.832	3	<0.001***
	LPS + Butyrate	15	104 (2 – 743)			
	Butyrate	15	4 (0.6 – 15)			
	Unstimulated	15	2 (1 – 8)			
IL-6	LPS	15	2285.32 (373.66 – 8034.47)	42.013	3	<0.001***
	LPS + Butyrate	15	1618 (8.85 – 6044.9)			
	Butyrate	15	49 (7 – 184.15)			
	Unstimulated	15	20.724 (3.55 – 79.38)			
Interferon-γ	LPS	15	31.52 (6.12 – 584.63)	20.018	3	<0.001***
	LPS + Butyrate	15	13.93 (6.12 – 29.57)			
	Butyrate	15	8.07 (1.27 – 25.66)			
	Unstimulated	15	10.02 (1.27 – 19.80)			
IL-13	LPS	15	254.381 (12.44 – 625.13)	4.511	3	0.211
	LPS + Butyrate	15	66.286 (8.26 – 328.18)			
	Butyrate	15	61.194 (8.25 – 454.08)			
	Unstimulated	15	61.194 (12.44 – 259.42)			
IL-10	LPS	15	50.909 (15.85 – 365.6)	42.247	3	<0.001***
	LPS + Butyrate	15	19 (4.46 – 59.49)			
	Butyrate	15	3 (0.3 – 4.38)			
	Unstimulated	15	3.62 (0.3 – 24.09)			
Poorly Controlled Type 2 Diabetes Mellitus						
TNF-α	LPS	15	1030 (301 – 4235)	47.725	3	<0.001***
	LPS + Butyrate	15	208 (27 – 2461)			
	Butyrate	15	3 (1 – 34)			
	Unstimulated	15	3 (1 – 7)			
IL-6	LPS	15	3185.405 (1215.6 – 7591.23)	47.010	3	<0.001***
	LPS + Butyrate	15	1299.974 (189.2 – 16554.46)			
	Butyrate	15	36.752 (10.44 – 351.70)			
	Unstimulated	15	21.737 (3.73 – 110.93)			
Interferon-γ	LPS	15	33.48 (8.07 – 178.11)	19.716	3	<0.001***
	LPS + Butyrate	15	14.19 (1.27 – 295.37)			
	Butyrate	15	8.07 (1.27 – 19.80)			
	Unstimulated	15	11 (1.27 – 28.89)			
IL-13	LPS	15	329.319 (24.78 – 731.42)	7.287	3	0.063
	LPS + Butyrate	15	121.426 (16.52 – 517.07)			
	Butyrate	15	121.426 (8.26 – 675)			
	Unstimulated	15	97.505 (8.26 – 315.86)			
IL-10	LPS	15	60.931 (14.55 – 258.00)	45.945	3	<0.001***
	LPS + Butyrate	15	22.437 (8.45 – 67.89)			
	Butyrate	15	2.838 (0.3 – 5.62)			
	Unstimulated	15	2.208 (0.3 – 16.75)			

TNF-α, tumor necrosis factor-α; IL-6, interleukin-6; IL-13, interleukin-13; IL-10, interleukin 10; LPS, lipopolysaccharide at total concentration of 100 ng/mL; LPS + butyrate, lipopolysaccharide at total concentration of 100 ng/mL and sodium butyrate at total concentration of 1 mM; and butyrate, sodium butyrate stimulation at total concentration of 1 mM; df, degree of freedom. The comparisons were assessed using the Kruskal–Wallis test. The data are presented as median (minimum–maximum) pg/mL. ***p < 0.001.

Compared to PBMC culture stimulated with LPS alone, treatment with sodium butyrate in LPS-stimulated culture suppressed only pro-inflammatory cytokines—TNF-α (*p = *0.039) and IFN-γ (*p* = 0.038)—in normoglycemic participants. Interestingly, although cytokine production from LPS stimulated culture in participants with poorly controlled T2DM was similar to that of normoglycemic participants, treatment with 1 mM sodium butyrate did not significantly reduce TNF-α (*p = *0.062) or IFN-γ (*p = *0.065) production.

Without LPS stimulation, treatment with sodium butyrate was unable to stimulate or suppress PBMC culture, which was shown by the similar amount of cytokine production in the unstimulated PBMC culture and the culture stimulated with sodium butyrate alone (**Figure 2**). Moreover, sodium butyrate treatment did not affect the production of IL-6, IL-13, and IL-10 by LPS-stimulated culture either in the normoglycemic or poorly controlled T2DM group (**Figures 2B, D, E**).

## Discussion

T2DM is classified as a low-grade inflammatory condition (12). Low-grade inflammatory condition is closely related to altered intestinal microbiota or dysbiosis (18, 19). Dysbiosis alters SCFA production in T2DM, thus making the body vulnerable to inflammation (20). Moreover, several studies have revealed that translocation of gut microbiota, especially endotoxins and LPS, worsens inflammation in T2DM (21, 22).

Butyrate is the most potent SCFA that exerts anti-inflammatory properties. About 95% of butyrate is absorbed from the intestinal lumen (23). However, the plasma butyrate level is far lower than the intracolonic butyrate level because colonocytes use butyrate as an energy source. In our study, the plasma butyrate concentration was in the range of 0.1–0.8 mM in participants with poorly controlled T2DM and 0.1–0.7 mM in normoglycemic participants. These concentrations are higher than that of healthy normoglycemic western population, which has a concentration in the range of 0.01–0.1 mM (24, 25). This finding might be influenced by the Asian diet, which is relatively low in meat, dairy products, and sugar but high in vegetables and resistant starches. We also found that plasma butyrate did not differ between poorly controlled T2DM and normoglycemic participants. Contrary to our finding, Muller et al. (26) showed that fasting circulating butyrate was negatively associated with fasting glucose and free fatty acid levels but was not correlated with inflammatory markers in obese, non-diabetes participants. Nishitsuji et al. (20) also found that although total SCFA levels were lower in obese diabetic mice than in non-obese mice, the plasma butyrate levels of obese diabetic mice were higher, which was correlated with an increased ratio of Gram-positive to Gram-negative gut microbiota.

Nancey et al. (27) reported that the anti-inflammatory effect of butyrate was achieved when butyrate was given in concentrations higher than the physiological level. In our study, we cultured PBMC in the presence of 1 mM sodium butyrate. In normoglycemic participants, sodium butyrate significantly reduced the LPS-stimulated release of TNF-α but not IL-6. Segain et al. (9) reported that the LPS-induced translocation of NF-kB was inhibited by sodium butyrate. Therefore, butyrate treatment downregulated the LPS-stimulated mRNA expression of TNF-α, TNF-β, IL-6, and IL-1β. Similarly to our result, Nancey et al. (27) demonstrated that although TNF-α and IL-6 were encoded by the same transcription factor, NF-kB, sodium butyrate (0.0625–2 mM) was found to inhibit the LPS + PHA–stimulated release of TNF-α but not IL-6. Moreover, butyrate did not affect IL-6 production induced by *Mycobacterium tuberculosis* (28). Butyrate also had an inhibitory effect on Th-1 cell activity, marked by a reduced release of IFN-γ in normoglycemic subjects, but sodium butyrate did not affect the anti-inflammatory activity of Th-2 cytokines (IL-13 and IL-10) in either group. In line with this result, and despite the possibility that IL-10 would act as a mediator of butyrate’s anti-inflammatory effect, Lachmandas et al. (28) reported that SCFAs, including butyrate, also failed to affect LPS-induced IL-10 production. The presence of such conflicting results even in normoglycemic or healthy participants suggests that the mechanism by which sodium butyrate affects cytokine production is complex and warrants further study.

Interestingly, in the PBMC of participants with poorly controlled T2DM, 1 mM butyrate treatment did not affect LPS-stimulated cytokine release, either innate or adaptive, in contrast to findings in normoglycemic participants. Cleophas et al. (29) found that four-week oral sodium butyrate supplementation in participants with metabolic syndrome showed that oral sodium butyrate did not influence the cytokine-producing capacity of PBMC assessed by measuring IL-1β, IL-1Ra, IL-6, and IL-10 upon bacterial stimulation. We can only speculate that our sodium butyrate concentration was inadequate to inhibit HDAC activity or that PBMCs from participants with poorly controlled T2DM have an impaired response to sodium butyrate treatment. The failure of sodium butyrate treatment was also seen in ulcerative colitis patients: Magnusson et al. (30) reported that sodium butyrate was more potent in down-regulating inflammatory gene expression in non-inflamed healthy tissue than in the inflamed tissue of ulcerative colitis patients.

Some limitations of our study need to be addressed. First, we studied the effects of a single concentration of sodium butyrate on LPS-stimulated cytokine release in PBMCs. As the 1 mM sodium butyrate only partly affected cytokine release in normoglycemic and poorly controlled T2DM participants, gene expression and the dose-effect relationship should be studied to elaborate our findings. Second, we did not compare the plasma butyrate and cytokine production of participants with well-controlled T2DM to those with metabolic syndrome to investigate the time when the cytokine-producing capacity of PBMC is impaired. Third, these results do not reflect the *in vivo* condition in participants with poorly controlled T2DM because the duration of diabetes, micro- and macrovascular complications, and various diabetes medications could interfere with the intestinal microbiota and immune responses. However, these findings may at least partly explain why high plasma butyrate levels are not adequate to maintain an anti-inflammatory response in T2DM, particularly in poorly controlled T2DM.

## Conclusion

Plasma butyrate levels in participants with poorly controlled T2DM were not significantly different from those in normoglycemic participants. Compared to treatment with an LPS-stimulated PBMC culture, treatment with 1 mM sodium butyrate reduced the levels of TNF-α (p < 0.039) and IFN-γ (p < 0.038) in normoglycemic participants. The same general trend was seen in PBMC from participants with poorly controlled T2DM, but higher variability appeared to preclude statistical significance. These data suggest that sodium butyrate may modulate inflammatory cytokine production in human PBMCs but more research is needed to determine if butyrate is anti-inflammatory in poorly controlled T2DM.

## Data Availability Statement

The raw data supporting the conclusions of this article will be made available by the authors, without undue reservation.

## Ethics Statement

The studies involving human participants were reviewed and approved by the ethical committee of the Faculty of Medicine from Universitas Indonesia with reference number 18-03-0236. The patients/participants provided their written informed consent to participate in this study.

## Author Contributions

HW and DS contributed to conception, design, and acquisition; critically drafted the manuscript; revised the manuscript; gave final approval; and agreed to be accountable for all aspects of work ensuring integrity and accuracy. DT and RK contributed to acquisition, critically drafted the manuscript, revised the manuscript, gave final approval, and agreed to be accountable for all aspects of work ensuring integrity and accuracy. SP and RL contributed to interpretation, critically revised the manuscript, gave final approval, and agreed to be accountable for all aspects of work ensuring integrity and accuracy. All authors contributed to the article and approved the submitted version.

## Funding

This research was supported by Universitas Indonesia Research Grant (PUTI) Q2 with reference number NKB-4084/UN2.RST/HKP.05.00/2020.

## Conflict of Interest

The authors declare that the research was conducted in the absence of any commercial or financial relationships that could be construed as a potential conflict of interest.

## Publisher’s Note

All claims expressed in this article are solely those of the authors and do not necessarily represent those of their affiliated organizations, or those of the publisher, the editors and the reviewers. Any product that may be evaluated in this article, or claim that may be made by its manufacturer, is not guaranteed or endorsed by the publisher.
