# Supplementary material for: Impact of Sodium Butyrate Treatment in LPS-Stimulated Peripheral Blood Mononuclear Cells of Poorly Controlled Type 2 DM
Source: Front Endocrinol (Lausanne). 2021 Jul 29;12:652942. doi: 10.3389/fendo.2021.652942 (PMC8358792; doi:10.3389/fendo.2021.652942)
Supplement: Supplementary file 1 [file DataSheet_1.pdf]

Supplemental Table 1. Mann-Whitney test comparing the production of TNF-α, IL-6, interferon-γ, IL-13, and IL-10 between normoglycemic subjects and poorly controlled T2DM patients in LPS, LPS + butyrate, butyrate stimulated PBMC cultures, and unstimulated PBMC cultures

Cytokines	Subject	n	Median (minimum-maximum)	*p*-value
LPS-stimulated PBMC culture				
TNF-α	Normoglycemic	15	707 (73 – 4071)	0.126
	Poorly controlled-T2DM	15	1030 (301 – 4235)	
IL-6	Normoglycemic	15	2285.32 (373.66 – 8034.47)	0.806
	Poorly controlled-T2DM	15	3185.40 (1215.6 – 7591.23)	
Interferon-γ	Normoglycemic	15	31.52 (6.12 – 584.63)	0.935
	Poorly controlled-T2DM	15	33.48 (8.07 – 178.11)	
IL-13	Normoglycemic	15	254.38 (12.44 – 625.13)	0.313
	Poorly controlled-T2DM	15	329.319 (24.78 – 731.42)	
IL-10	Normoglycemic	15	50.909 (15.85 – 365.6)	0.683
	Poorly controlled-T2DM	15	60.931 (14.55 – 258)	
LPS + Butyrate-stimulated PBMC culture				
TNF-α	Normoglycemic	15	104 (2 – 743)	0.137
	Poorly controlled-T2DM	15	208 (27 – 2461)	
IL-6	Normoglycemic	15	1618 (8.85 – 6044.9)	0.806
	Poorly controlled-T2DM	15	1299.97 (189.2 – 16554.46)	
Interferon-γ	Normoglycemic	15	13.93 (6.12 – 29.57)	0.806
	Poorly controlled-T2DM	15	14.91 (1.27 – 295.37)	
IL-13	Normoglycemic	15	66.286 (8.26 – 328.18)	0.232
	Poorly controlled-T2DM	15	121.426 (16.52 – 517.07)	
IL-10	Normoglycemic	15	19 (4.46 – 59.49)	0.806
	Poorly controlled-T2DM	15	22.437 (8.45 – 67.89)	
Butyrate-stimulated PBMC culture				
TNF-α	Normoglycemic	15	4 (0.6 – 15)	0.653
	Poorly controlled-T2DM	15	3 (1 – 34)	
IL-6	Normoglycemic	15	49 (7 – 184.15)	0.806
	Poorly controlled-T2DM	15	36.752 (10.44 – 351.7)	
Interferon-γ	Normoglycemic	15	8.07 (1.27 – 25.66)	0.870
	Poorly controlled-T2DM	15	8.07 (1.27 – 19.80)	
IL-13	Normoglycemic	15	61.194 (8.25 – 454.08)	0.738
	Poorly controlled-T2DM	15	121.426 (8.26 – 675)	

IL-10	Normoglycemic	15	3 (0.3 – 4.38)	0.713
	Poorly controlled-T2DM	15	2.838 (0.3 – 5.62)	
Unstimulated PBMC culture				
TNF-α	Normoglycemic	15	2 (1 – 8)	0.389
	Poorly controlled-T2DM	15	3 (1 – 7)	
IL-6	Normoglycemic	15	20.724 (3.55 – 79.38)	0.870
	Poorly controlled-T2DM	15	21.737 (3.73 – 110.93)	
Interferon-γ	Normoglycemic	15	10.02 (1.27 – 19.8)	0.653
	Poorly controlled-T2DM	15	11 (1.27 – 28.89)	
IL-13	Normoglycemic	15	61.194 (12.44 – 259.42)	0.927
	Poorly controlled-T2DM	15	97.505 (8.26 – 315.86)	
IL-10	Normoglycemic	15	3.624 (0.3 – 24.09)	0.106
	Poorly controlled-T2DM	15	2.208 (0.3 – 16.75)	

Comparisons of the production of TNF-α, IL-6, interferon-γ, IL-13, and IL-10 between normoglycemic subjects and poorly controlled T2DM patients in LPS, LPS + butyrate, butyrate stimulated PBMC cultures, and unstimulated PBMC cultures. T2DM: type 2 diabetes mellitus; TNF-α: tumor necrosis factor-α; IL-6: interleukin-6; IL-13: interleukin-13; IL-10: interleukin 10; LPS: lipopolysaccharide at total concentration of 100 ng/mL; LPS + butyrate: lipopolysaccharide at total concentration of 100 ng/mL and sodium butyrate at total concentration of 1 mM; and butyrate: sodium butyrate stimulation at total concentration of 1 mM. The comparisons were assessed using the Mann-Whitney U test. The data were presented as median (minimum-maximum) pg/mL.

Supplemental table 2. Bonferroni Adjustment for six comparisons of the production of TNF-α, IL-6, interferon-γ, and IL-10 in the PBMC of normoglycemic participant and poorly controlled type 2 diabetes mellitus

Cytokines	Comparing groups	Test Statistics	Standard Error	Adjusted *p*-value
Normoglycemic Participants				
TNF-α	LPS – LPS + Butyrate	13.1	6.36	0.039*
	LPS – Butyrate	33.167		< 0.001***
	LPS – Unstimulated	37.6		< 0.001***
	LPS + Butyrate –Butyrate	20.067		0.002**
	LPS + Butyrate – Unstimulated	24.5		< 0.001***
	Butyrate – Unstimulated	4.433		0.486
IL-6	LPS – LPS + Butyrate	6.333	6.377	1
	LPS – Butyrate	28.533		< 0.001***
	LPS – Unstimulated	34.867		< 0.001***
	LPS + Butyrate –Butyrate	22.2		< 0.001***
	LPS + Butyrate – Unstimulated	28.533		< 0.001***
	Butyrate – Unstimulated	6.333		1
Interferon-γ	LPS – LPS + Butyrate	13.2	6.364	0.038*
	LPS – Butyrate	25.233		< 0.001***
	LPS – Unstimulated	23.567		< 0.001***
	LPS + Butyrate –Butyrate	12.033		0.059
	LPS + Butyrate – Unstimulated	10.367		0.103
	Butyrate – Unstimulated	-1.667		1
IL-10	LPS – LPS + Butyrate	9	6.374	0.158
	LPS – Butyrate	35.267		< 0.001***
	LPS – Unstimulated	30.533		< 0.001***
	LPS + Butyrate –Butyrate	26.267		< 0.001***
	LPS + Butyrate – Unstimulated	21.533		0.001**
	Butyrate – Unstimulated	-4.733		1
Poorly controlled Type 2 Diabetes Mellitus				
TNF-α	LPS – LPS + Butyrate	11.867	6.364	0.062
	LPS – Butyrate	35.633		< 0.001***
	LPS – Unstimulated	36.1		< 0.001***
	LPS + Butyrate –Butyrate	23.767		< 0.001***
	LPS + Butyrate – Unstimulated	24.233		< 0.001***
	Butyrate – Unstimulated	0.467		1
IL-6	LPS – LPS + Butyrate	8	6.377	0.21
	LPS – Butyrate	30.2		< 0.001***
	LPS – Unstimulated	37.667		< 0.001***
	LPS + Butyrate –Butyrate	22.2		< 0.001***
	LPS + Butyrate – Unstimulated	29.667		< 0.001***
	Butyrate – Unstimulated	7.467		0.242

Interferon-γ	LPS – LPS + Butyrate	11.733	6.367	0.065
	LPS – Butyrate	26.133		< 0.001***
	LPS – Unstimulated	21.333		0.001**
	LPS + Butyrate –Butyrate	14.4		0.024*
	LPS + Butyrate – Unstimulated	9.6		0.132
	Butyrate – Unstimulated	-4.8		1
IL-10	LPS – LPS + Butyrate	10.433		0.102
	LPS – Butyrate	34.133		< 0.001***
	LPS – Unstimulated	35.567		< 0.001***
	LPS + Butyrate –Butyrate	23.7		< 0.001***
	LPS + Butyrate – Unstimulated	25.133		< 0.001***
	Butyrate – Unstimulated	1.433		1

Multiple comparisons of the production of TNF-α, IL-6, interferon-γ, and IL-10 among LPS, LPS + butyrate, butyrate stimulated PBMC cultures, and unstimulated PBMC cultures. TNF-α: tumor necrosis factor-α; IL-6: interleukin-6; IL-10: interleukin 10; LPS: lipopolysaccharide at total concentration of 100 ng/mL; LPS + butyrate: lipopolysaccharide at total concentration of 100 ng/mL and sodium butyrate at total concentration of 1 mM; and butyrate: sodium butyrate stimulation at total concentration of 1 mM. The comparisons were assessed using the post hoc pairwise comparison. *$p < 0.05$; **$p < 0.01$; ***$p < 0.001$
